# Navigating Ethical Challenges in CAR-T Treatment

**DOI:** 10.1007/s10730-025-09574-5

**Published:** 2026-02-12

**Authors:** Elise R. A. Pennings, Anne M. Spanjaart, Anne Ruth Schaaphok, Marjolein A. van Maanen, Sanne H. Tonino, Maria T. Kuipers, Marie José Kersten, Suzanne Metselaar

**Affiliations:** 1 Department of Hematology, Amsterdam UMC Location University of Amsterdam, Amsterdam, The Netherlands; 2 Cancer Center Amsterdam, Amsterdam, The Netherlands; 3 LYMMCARE (Lymphoma and Myeloma Center Amsterdam), Amsterdam, The Netherlands; 4 Erasmus School of Health Policy and Management, Erasmus University Rotterdam, Rotterdam, The Netherlands; 5 Department of Internal Medicine, Section of Geriatric Medicine, Amsterdam UMC Location University of Amsterdam, Amsterdam, The Netherlands; 6 Department of Hematology, Amsterdam UMC Location Vrije Universiteit Amsterdam, Amsterdam, The Netherlands; 7 Department of Ethics, Law, and Humanities, Amsterdam UMC Location VUmc, Amsterdam, The Netherlands

**Keywords:** CAR T-cell therapy, Ethical challenges, Clinical ethics support, Moral case deliberation

## Abstract

Chimeric Antigen Receptor T-cell (CAR-T) therapy has unprecedentedly improved outcomes for patients with relapsed or refractory B-cell malignancies, offering the prospect of survival to patients with otherwise poor prognoses. Despite these advances, CAR-T therapy presents multiple challenges as it is not always effective, possibly harmful with severe, potentially life-threatening side effects, resource-demanding and expensive. Furthermore, patient selection and prioritization (e.g., in case of limited hospital or manufacturing capacity) is complex, as no standardized criteria exist and reliable predictive tools to determine individual outcomes upfront are still being developed. This creates ethical challenges that healthcare professionals need to navigate in order to provide good care to their patients, and that may be a cause of moral distress, which may affect their well-being. Addressing these ethical challenges is therefore crucial for both patients and healthcare professionals. Yet, up until now, they are hardly explored in the context of CAR-T treatment. We will therefore present a preliminary overview of current ethical challenges in CAR-T treatment in clinical practice, based on our experiences as clinicians and clinical ethicists. Furthermore, we will address how these challenges may be navigated in clinical practice, proposing clinical ethics support, particularly through moral case deliberation. As the application of CAR-T therapy is increasing, it is expected that more healthcare professionals will encounter ethical challenges and this warrants adequate support to enhance quality of patient care while also contributing to the well-being of healthcare professionals.

## Introduction

Chimeric Antigen Receptor T-cell (CAR-T) therapy has revolutionized the treatment of patients with relapsed or refractory (R/R) B-cell malignancies. Prior to the introduction of CAR-T therapy, these patients had limited treatment options in the R/R setting and had a very poor prognosis. For example, with previous treatments, R/R large B-cell lymphoma (LBCL) patients had poor survival (median overall survival: 6.3 months, 2-year overall survival rate: 20%), with only few patients achieving a complete response (7%) (Crump et al., 2017). However, with CAR-T therapy (axicabtagene ciloleucel; axi-cel) complete responses are achieved in 58% of patients and durable remissions are seen with an estimated 5-year progression-free and overall survival rate of 32% and 43%, respectively (Neelapu et al., 2023). Despite its curative potential, CAR-T therapy is not effective in all patients and poses several challenges. Therefore, the question whether or not to offer CAR-T therapy to an individual patient does not always have an easy and straightforward answer. See Box 1 for a detailed description of CAR-T treatment and its challenges.

**Box 1:** Description of CAR T-cell therapy

Standard of care CAR-T treatment consists of a single infusion of autologous derived T-cells, which have been genetically modified to express a chimeric antigen receptor (CAR). This CAR T-cell combines the cytolytic power of a T-cell with an antibody-based specificity for an antigen on tumor cells (e.g., CD19 on B-cells).
Although generally only one infusion is needed, the treatment trajectory as a whole is complex and intensive. When deemed potentially eligible, patients enter a screening phase to confirm eligibility.
Thereafter, the apheresis of T-cells takes place, after which the cells enter a manufacturing phase, also referred to as the bridging phase (median duration of 4–6 weeks). During this bridging phase, patients may receive bridging therapy. Eligible patients are often refractory or relapsed, with an aggressive, and fast growing disease, which is generally unresponsive to conventional therapy. Therefore, it can be difficult to successfully bridge patients during the manufacturing time, especially in case of highly aggressive disease. CA-T therapy is often a last resort for these patients but not always feasible. Prior to CAR-T infusion, patients start receiving conditioning chemotherapy for three days. After infusion, patients are often hospitalized for approximately 7 days at minimum and in case of severe toxicity patients need to be admitted to ICU. After discharge up to 4 weeks post-infusion, 24/7 presence of an informal caregiver is required to monitor acute toxicity. Subsequently, up to 15 years post-infusion, long-term follow-up is required to monitor long-term toxicity, response and survival. The most common CAR-T related acute toxicities include cytokine release syndrome (CRS) and immune effector-cell associated neurotoxicity syndrome (ICANS). CRS is an inflammatory syndrome which most commonly manifests with constitutional symptoms such as fever and malaise but can affect all organs with multi-organ failure in severe cases. ICANS is also an inflammatory syndrome characterized by encephalopathy with altered mental status including confusion, aphasia, and somnolence, and may progress into epileptic seizures, focal neurological signs such as motor weakness, and loss of consciousness (Neelapu et al., 2018). In addition, ongoing toxicities that may be acute but also persist on the long-term are immune effector-cell associated hematotoxicity (ICAHT) and infections due to impaired immune reconstitution (Rejeski et al., 2023, Rejeski et al., 2025). Also, non-classical neurological toxicity (other than ICANS), such as movement and neurocognitive toxicity, is more often reported and can have a substantial impact (Graham et al., 2025). Furthermore, CAR-T therapy is associated with a higher risk of secondary malignancies such as myelodysplastic syndrome/ acute myeloid leukemia. Twelve-month non-relapse mortality after CAR-T infusion across different CAR-T products and B-cell malignancies is 7% and mainly driven by infections (Cordas dos Santos et al., 2024). CAR-T therapy is among the most expensive therapies currently reimbursed with costs ranging between ± $400,000–500,000 per patient and places a significant strain on scarce healthcare resources (Hernandez et al., 2018; June & Sadelain, 2018; Sureda et al., 2023).
As CAR-T therapy is a relatively novel treatment, evidence about many aspects is still being gathered and prognostic and predictive models are still in development. Therefore, treatment outcomes, such as the effectiveness and the severity of side-effects in different patient subgroups, can be hard to predict upfront.

*CAR-T* Chimeric antigen receptor T-cell, *CRS* Cytokine release syndrome, *ICAHT* immune effector-cell associated hematotoxicity, *ICANS* Immune effector cell-associated neurotoxicity syndrome, *ICU * Intensive care unit

The unique aspects and complexities of CAR-T treatment, including its high costs, demands on healthcare resources, and distinctive toxicity profile with potentially life-threatening side effects, make patient selection both essential and challenging. Moreover, when hospital capacity, CAR T manufacturing slots or clinical trial availability is limited, patients need to be prioritized. Currently, clinical tools to predict, at time of patient selection, the efficacy and safety outcomes in individual patients, are still being developed. While the in- and exclusion criteria used in the clinical trials provide some guidance to clinicians to select patients for CAR-T therapy, no standardized selection procedures with strict eligibility criteria for the real world population exist. For support, clinicians often collaborate at the local and/or national level (e.g., in multidisciplinary teams or tumor boards) to jointly decide whether to treat a patient with CAR-T therapy and to collectively establish eligibility criteria. However, these criteria vary across and even within countries and continuously evolve over time as more experience is gained with CAR-T therapy. Furthermore, although these criteria provide some guidelines, they are not strict and exhaustive checklists, hence areas of uncertainty persist and clinicians may still face ambiguity regarding the best course of action (Bell et al., 2023; Kourelis et al., 2023; Spanjaart et al., 2023). All in all, clinicians providing CAR-T therapy regularly face ethical challenges when selecting and prioritizing patients, i.e., situations in which the best course of action and/or what is " good care" for a patient is not obvious, and which are therefore experienced as troublesome (Bell et al., 2023; Imbach et al., 2018; Kourelis et al., 2023).

Recognizing and effectively addressing these ethical dilemmas is important, as it not only enhances patient care and fosters fair allocation of resources, but also improves the well-being of healthcare professionals by supporting them in dealing with moral distress. Moral distress can be defined as the mental burden caused by the experience of an ethical challenge and is related to a plethora of negative emotions, such as sadness, frustration, feelings of powerlessness, and anxiety (Morley et al., 2019). Previous studies have shown that unresolved moral distress can contribute to decreased quality of care, higher employee turn-over, sick leaves, decreased job satisfaction, and burn-out (Bodenheimer & Sinsky, 2014; de Veer et al., 2013; Lamiani et al., 2017; McHugh et al., 2011; Molewijk et al., 2015). Therefore, providing adequate support, such as clinical ethics support (CES) to help clinicians navigate ethical challenges in CAR-T therapy, is important.

However, up until now, studies providing a broad exploration of ethical challenges, as well as closer analyses of how healthcare professionals can be supported in dealing with specific ethical issues around CAR-T treatment, are scarce. As healthcare professionals working with CAR-T treatment at a hematology ward of an academic hospital (ERAP, AMS, MAvM, SHT, MTK, MJK), and as ethicists providing clinical ethics support to healthcare professionals (ARS, SM), we therefore present a first exploration of frequently encountered ethical challenges in CAR-T treatment in clinical practice. Although these issues are often interconnected in concrete cases, we discuss them consecutively for reasons of clarity. Furthermore, we illustrate how ethics support can be implemented in CAR-T clinical practice and support clinicians in navigating their ethical challenges. Specifically, we will discuss moral case deliberation (MCD), a dialogical approach to clinical ethics support. By raising awareness of the ethical complexities associated with CAR-T therapy, highlighting the usefulness of applying tools as MCD to provide adequate support and underlining the value of clinicians collaborating with ethicists, this study seeks to further improve CAR-T care while safeguarding the well-being of the medical team.

## Ethical Challenges

### Non-Maleficence

One major area of ethical concern is the possibility of CAR-T related toxicity to outweigh therapeutic benefits, raising questions for physicians about adherence to the principle of *non-maleficence*. The “one and done” nature of CAR-T treatment, in which a single infusion with living CAR T-cells with persistence and renewal capacity can provide sustained benefit and even cure, is remarkable. But, this unique characteristic also renders it a definitive intervention that cannot be reversed once administered. This is particularly concerning because it can induce severe and potentially life-threatening toxicities such as cytokine release syndrome (CRS), neurotoxicity, infections, and immune effector-cell associated hematotoxicity (ICAHT), which can significantly impact both patients’ survival and quality of life, necessitates intensive monitoring and can result in frequent hospital visits and re-admissions (Cappell & Kochenderfer, 2023; June & Sadelain, 2018; Rejeski et al., 2023; Spanjaart et al., 2022; Sureda et al., 2023).

What if CAR-T therapy is indicated, but doubts remain out of fear that the side effects might harm the patient more than (s)he will benefit from the treatment itself? Which (risk and severity of) harm is justified vis-à-vis the (possible) benefits of treatment with CAR-T therapy? These questions are particularly pertinent in patients with rapidly progressive disease or underlying comorbidities, in whom the risk of side effects may be increased and/or the chance to respond well to the treatment is reduced.

For example, consider a 40-year old patient, a mother of three young children, diagnosed with rapidly progressive LBCL, not responding to all prior therapy lines, for whom CAR-T therapy is the last curative treatment option. However, awaiting her CAR-T cells being manufactured, the LBCL does not respond to bridging therapy, resulting in a high tumorload and consequently a poor performance status. While the patient is still determined to receive the CAR-T cells, the question arises whether it is still justified to proceed, given the low likelihood to respond and the high risk of severe side effects. What if she dies being severely confused and somnolent due to neurotoxicity while admitted at the intensive care unit without having had the chance to properly say goodbye to her husband and children?^1^

Doubts may also arise due to the tension between the promise of CAR-T therapy and the possibility of iatrogenic harm in the case of frail patients due to old age and comorbidities. In practice, all patients with LBCL who meet the eligibility criteria can receive CAR-T therapy (as ≥ third-line treatment) in the Netherlands; there is no age limit (Spanjaart et al., 2023). Patients up to 90 years treated with CAR-T therapy have been reported in literature (Chihara et al., 2023). However, frequently, moral dilemmas occur. Should you provide CAR-T treatment to an octogenarian patient who already has mild cognitive impairment, when side effects, especially on cognitive functioning, may be significant? Especially if this patient expresses a strong wish to be at home as long as possible and care for his wife who needs support on a daily basis?

Most studies show similar or even better response rates and survival in older patients compared to younger patients. While 30% of the patients are older than 75 years when diagnosed with LBCL, studies evaluating outcomes in patients  < 75 versus patients  ≥ 75 years are scarce. Primarily a threshold of  < 65 and  ≥ 65 is used, which makes it difficult to estimate the outcomes of CAR-T therapy in much older patients (Kim & Chihara, 2025; Sehn & Salles, 2021; Shune et al., 2025). One study that did evaluate patients  ≥ 75 years, reported a significantly shorter event-free survival compared to patients aged 65–74 years, while another study did not find an association of age  ≥ 75 years with survival outcomes (Chihara et al., 2023; Johnson et al., 2024). Outcomes regarding toxicity in older compared to younger patients vary, with some studies showing no differences and other studies reporting higher and more severe toxicity, in particular neurotoxicity, in older patients compared to younger patients (Johnson et al., 2024; Kim & Chihara, 2025; Shune et al., 2025). The long-term effects of toxicity, such as the effect of neurotoxicity on cognitive functioning in older patients remains uncertain, but is a matter of concern (Shune et al., 2025). The question whether to offer CAR-T therapy can be difficult in case alternative therapies are available that may not cure the patient, but which will help with symptom management and improvement of quality of life for a significant period of time, without the risk of significant side effects.

### Navigating Uncertainty

Compounding the challenge to adhere to the principle of non-maleficence is the lack of robust clinical evidence on the safety and efficacy of CAR-T therapy. Specific patient subgroups were excluded from pivotal clinical trials, such as patients with HIV infection and central nervous system disorders. Consequently, treatment outcomes, including therapeutic efficacy and the likelihood or severity of adverse effects in these patient subgroups, were unknown. Also, as a relatively new therapeutic modality, CAR-T therapy is still undergoing clinical and scientific evaluation and prognostic and predictive models are still being developed. As a result, clinicians often must make decisions in the absence of high-quality evidence, further complicating the risk–benefit assessment and intensifying ethical doubts. This calls for a careful integration of ethical reflection alongside empirical reasoning, in which recognizing the limits of evidence and attending to the values, preferences, and broader context of individual patients is essential. For example, consider a 50-year old patient with HIV and a diagnosis of R/R LBCL. When there is limited evidence on the efficacy and safety of CAR-T treatment in patients with HIV, should you take the risk to provide CAR-T treatment? Especially as this is also a possibility to acquire more experience in this patient subgroup?

### Shared Decision-Making and Appropriate Care

Also in a more fundamental way, physicians providing CAR-T therapy are confronted with ethical challenges in determining what constitutes the most appropriate care for their individual patients, i.e., care that aligns optimally with what they deem important and valuable. Currently, for some patients with relapsed or refractory (R/R) B-cell malignancies CAR-T therapy represents the only potentially curative option. In this regard, it is often considered the best available treatment. However, in specific cases, questions may arise as to whether it is also the best choice for an individual patient. For certain patients, prioritizing the best possible quality of life—for instance including the opportunity to remain at home, surrounded by loved ones—may be more appropriate and constitute better care than pursuing aggressive hospital-based treatment aimed primarily at prolonging life (Kaur & Mohanti, 2011; Schofield et al., 2006).

These concerns are closely tied to the broader issue of when to shift the focus of care from curative to palliative intent. The decision to transition from curative to palliative care is a nuanced one, requiring careful ethical deliberation that takes into account not only clinical indicators but also the patient’s values and preferences, whether that be the possibility of cure or the preservation of comfort, dignity, or a certain way of life. A collaborative approach between clinicians and patients is essential to ensure that treatment decisions reflect both medical expertise and the patient’s individual goals and preferences.

To accomplish this, patients must be given clear, relevant, and comprehensible information about the potential benefits and risks associated with CAR-T therapy and alternatives in order to participate in meaningful shared decision-making. However, achieving this level of understanding can be particularly challenging in the context of a rapidly progressive and life-threatening illness, where time pressure and emotional distress may limit a patient’s capacity to fully absorb complex medical information. An additional layer of ethical complexity emerges in situations where there is uncertainty about the extent to which a patient comprehends what CAR-T therapy entails. Although a patient may express a clear desire to undergo CAR-T treatment and may formally meet the criteria for informed consent, concerns may remain as to whether the patient fully understands the burden and potential side effects associated with this therapy. This is particularly relevant given the novelty, complexity and intensity of the treatment, and the difficulty in predicting individual outcomes in advance.

### Distributive Justice

Another set of ethical concerns around CAR-T therapy relates to distributive justice—i.e., the fair allocation of limited healthcare resources. In the Netherlands, as in many other countries, CAR-T therapy is among the most costly treatments currently reimbursed, with prices ranging from approximately $400,000 to $500,000 per patient, and places a significant burden on increasingly scarce healthcare resources (Hernandez et al., 2018; June & Sadelain, 2018; Sureda et al., 2023). This introduces an ethical tension between what may be best for an individual patient and what is feasible or justifiable at the institutional or societal level, especially in publicly financed healthcare systems such as the Dutch system (Schofield et al., 2021). As the number of patients eligible for CAR-T therapy continues to grow, so too will the strain on budgets and infrastructure. More broadly, the high cost of CAR-T therapy raises questions about the sustainability of funding high-cost innovations in healthcare. This is not only a matter for policy-makers and hospital management; physicians are also increasingly confronted with the difficult realities of resource allocation in clinical practice. For example, when it is national policy that CAR-T therapy for patients with R/R multiple myeloma is not reimbursed in your country, should you refer your wealthy 50-year old patient with R/R multiple myeloma to a neighboring country where CAR-T therapy is reimbursed?

### Patient Selection and Prioritization

While more evidence on factors associated with outcomes after CAR-T therapy becomes available and are being included in predictive models, is it ethical to refuse CAR-T treatment to a 60-year old patient who has a strong wish to receive CAR-T therapy, but has a high risk score for toxicity? When can scores from risk stratification tools be used as formal exclusion criteria?

In practice, there are limited treatment slots for CAR-T-therapy. How to allocate patients in a justifiable way? Consider, for example, the scenario in which a 40-year-old patient is referred for CAR-T therapy after an 80-year-old patient, but due to limited treatment slots, the younger patient may be denied timely access. While helpful guidelines exist to offer some support for prioritization (Bell et al., 2023; Kourelis et al., 2023), these tools do not fully eliminate the ethical difficulty of making comparative judgments between patients, even when treatment prognoses or life expectancy differ. In this particular case, the physicians deemed interfering with the order of treatment of patients on a first come, first serve basis unfair. Furthermore, they argued, while prioritizing patients with the highest likelihood of responding to therapy and/or the longest survival expectancy may maximize overall treatment success and justify the high cost of care, it risks reinforcing inequities by marginalizing vulnerable patient groups such as elderly patients. Yet, they felt great concern for their 40-year-old patient, especially as he had a young family and was "running out of time", as his illness was rapidly progressing.

The unique aspects and complexities of CAR-T treatment that we discussed here, including its capacity to cure otherwise incurable patients, demands on healthcare resources, potentially life-threatening toxicities, and a current lack of validated clinical tools to predict, at time of patient selection, efficacy and safety outcomes after CAR-T therapy in individual patients, underscore the necessity of ethical reflection that goes beyond standard clinical protocols. These reflections should go beyond the principle to avoid harm; the issues discussed demand a careful and context-sensitive weighing of risks, benefits, and uncertainties, particularly in the case of patients for whom clinical experience and evidence are lacking. How to warrant patient autonomy in this evolving therapeutic space? Furthermore, navigating these dilemmas requires ongoing ethical reflection that consider both individual needs and collective responsibilities. This calls for supporting health care professionals in dealing with the moral distress and burden of having to navigate the ethical challenges that they are confronted with.

## Clinical Ethics Support for Healthcare Professionals Providing CAR-T Treatment

### Moral Case Deliberation

Moral case deliberation (MCD) is a dialogical form of clinical ethics support. It aims to create a constructive dialogue to better understand and deal with a concrete ethical challenge by delving into the moral doubts, experiences, viewpoints, and judgments of all stakeholders involved. Elucidating what makes the situation morally difficult and finding the right course of action are main goals (Widdershoven & Molewijk, 2016). Usually, the participants of an MCD are healthcare professionals, but occasionally also patients and their family members can participate. Several methods exist to perform an MCD. A frequently used method for MCD, which was also used in this study, is the dilemma method, in which an ethical issue is articulated as a moral dilemma with two mutually exclusive options for action. It consists of 10 steps, as presented in Table 1 (Stolper et al., 2016). MCD can be organized either before a decision needs to be made or action needs to be taken, or in retrospect, in order to reflect upon how an ethically challenging situation was dealt with. The former may help to provide good care in a situation that is perceived as ethically complex. The latter is useful in order to reduce moral distress and moral doubts that might still be lingering among those involved and provide an opportunity for moral learning and guidance for future situations.

**Table 1:** Steps of the Dilemma method for Moral Case Deliberation (Stolper et al., 2016)

Steps of the Dilemma method	Description of each step
Step 1. Introduction	The facilitator introduces the aim and the procedure of moral case deliberation (MCD).
Step 2. Presentation of the case	The case presenter introduces the case , focusing on the facts, the experience of the case presenter and the "moment of heat" (i.e., the specific moment where the presenter felt his/her dilemma most strongly).
Step 3. Formulating the moral question and the dilemma	The case presenter with help of the other participants and the facilitator makes the dilemma explicit and identifies the underlying moral questions. The facilitator asks clarifying questions such as “what makes you doubt or feel uneasy”? Also, negative consequences of choosing one of both options of the dilemma are formulated to illustrate what is at stake.
Step 4. Clarification in order to place oneself in the situation of the case presenter	The facilitator invites the participants to ask questions to the case presenter to (re)construct the situation. The aim of these questions should be to create a precise understanding of the situation and to foster the ability for the participants to empathize with the case presenter.
Step 5. Analyzing the case in terms of perspectives, values and norms	The participants identify all relevant stakeholders involved and formulate their perspectives (norms and values). Subsequently, all relevant stakeholders and their perspectives are listed, in order to understand the complexity of the case.
Step 6. Looking for alternatives	All participants brainstorm about (out-of-the-box) alternative possible courses or actions besides the two options of the dilemma. This might be useful for participants to help understand their considerations when answering the moral dilemma question.
Step 7. Making an individual choice and making one’s considerations explicit	The facilitator invites each participant to articulate a course of action and justify this, paying attention to possible harm that may come along with their choice: *A: It is morally justified that I choose option … (A, B or an alternative);* *B: Because of … (which perspective/value/norm?);* *C: Despite of … (which perspective/value/norm?);* *D: How can you limit the damage of the choice made under ‘C’;* *E: What do you need to act according your answer under ‘A’?.*
Step 8. Dialogical inquiry	Differences and similarities between the individual considerations of all participants are evaluated as part of a dialogical inquiry. This enhances understanding of each other’s perspectives and choices.
Step 9. Conclusion	The participants are asked to share insights which have been gained, sum up conclusions, and make a plan for action. Additionally, key principles for similar cases in future can be formulated.
Step 10. Evaluation	The facilitator performs a short evaluation of the deliberation with all participants; how did the participants experience the process and the result of this MCD and what may be adjusted in future deliberations?

### A Case Example

In order to illustrate how MCD provides ethics support in the case of moral challenges in CAR-T treatment, we will present a summary of an MCD at a hematology ward of an academic hospital. The case of Mr. Mulder (fictional name) is discussed here (Box 2). Eleven healthcare professionals participated, including a facilitator and a case presenter. The facilitator was an ethicist, and the case presenter was a hematologist in training. The other participants included CAR-T clinical trial coordinators and researchers, a trained CAR-T nurse, a geriatrician, and CAR-T treating hematologists. In order to warrant anonymity, we changed some details of the case.

**Box 2:** The case of Mr. Mulder

Mr. Mulder, an 80-year-old patient, was diagnosed with DLBCL two years earlier. He was treated with first-line chemoimmunotherapy, but had progressive disease. He was deemed too old for salvage chemoimmunotherapy followed by an autologous stem cell transplantation and therefore received chemoimmunotherapy with a milder toxicity profile which shortly resulted in a good response.
Subsequently, when the lymphoma relapsed, he was referred for CAR-T therapy (axicabtagene ciloleucel, the only CAR-T product commercially available for DLBCL in the Netherlands at that time). He had no comorbidities and did not use any chronic medication. Neurologic examination showed no abnormalities. He had a good performance status (WHO 1). He had previously worked as a healthcare professional, was married, and had children. He indicated that he did not want to lose his independence; his autonomy was very important to him. He also had a very strong wish to survive and described himself as vivacious. He received one cycle of chemoimmunotherapy as bridging therapy that resulted in stable disease, with lymphoma above and below the diaphragm and extranodal lesions in skin, bones, and spleen at time of CAR-T infusion. Five days after infusion, he developed fever diagnosed as CRS grade 1. After CRS was treated with tocilizumab and resolved, he developed ICANS grade 2 with difficulties in finding words, writing a sentence, ataxia and a myoclonic tremor. An MRI scan showed no abnormalities. After 5 days of high-dose dexamethasone the neurologic symptoms resolved, except for persistent mild ataxia and myoclonic tremor. At time of hospital discharge, he had to rest almost all day due to severe fatigue.
One month post-CAR-T response evaluation showed complete resolution of all tumor lesions. In the following two months he was admitted to the hospital 3 times (total admission duration 6 weeks) with word-finding difficulties, mild confusion, ataxia, and myoclonic tremor severely interfering with his daily activities. The symptoms were attributed to ongoing CAR-T related neurotoxicity. High-dose dexamethasone resulted in resolution of symptoms but when reducing dosage (< 10 mg) all symptoms worsened again, severely affecting his daily life. At the 3-month post-infusion evaluation, the PET-CT scan revealed relapse of DLBCL in an axillary lymph node that resolved after treatment with radiotherapy.
However, his general condition worsened further, now with new noticeable short-term memory problems. The geriatrician diagnosed him with mild cognitive impairment. The patient did not wish for any further examination to detect the underlying cause. A few weeks later the DLBCL relapsed again and he preferred not to be treated anymore. He died soon after.

*CAR-T* Chimeric antigen receptor T-cell, *CRS* Cytokine release syndrome, *DLBCL* diffuse large B-cell lymphoma, *ICANS* Immune effector cell-associated neurotoxicity syndrome, *MRI* Magnetic resonance imaging, *PET-CT* 2-Deoxy-2-[18F]fluoro-D-glucose–positron emission tomography and computed tomography

The aim of this particular MCD was to reflect upon a situation that had occurred earlier, as moral doubts were still present among staff members whether good care was provided to the patient. The participants indicated beforehand that they wanted to learn from the reflection about how to deal with similar cases in the future. The MCD took approximately 90 minutes. Informed consent was obtained to participate in the study, audio record the session, and for publication of the case example. The audio recording was used to support the analyses.

In retrospect, the team, and in particular the treating hematologist, experienced feelings of doubt whether CAR-T treatment (axi-cel) was indeed the best option for Mr. Mulder, since he experienced severe persisting neurological side effects leading to many and lengthy hospital admissions and loss of quality of life. Moreover, he only responded shortly and relapsed soon after CAR-T infusion. Maybe an alternative treatment, less expensive and less intensive, with a more favorable toxicity profile but with a lower/unknown chance of long-term remission, would have been a better option? However, is it ethical to withhold a potentially curative treatment if you cannot fully predict the risk of (irreversible and severe) toxicity and chance of efficacy of the therapy? With the help of the facilitator, the case presenter (a hematologist in training) therefore articulated her dilemma as follows: *Should we have treated Mr. Mulder with CAR-T therapy or should we have treated him with an alternative therapy?* She also identified her underlying moral doubts, which were shared by the team: Is it morally justified to withhold a potentially curative treatment when it is available, and when there are no clear clinical factors or evidence indicating that it will not be effective for this particular patient? Otherwise, is it morally justified to risk harming this 80-year old patient with potential severe and long-lasting toxicity, which may severely impair his autonomy and quality of life, when it is uncertain whether he will achieve a durable response to the therapy? (See Box 3 for real world outcomes of patients with R/R LBCL treated with axi-cel after at least two prior therapy lines in the Netherlands). Compounding these doubts is the possibility that choosing to treat this patient with CAR-T therapy may delay or preclude treatment of other patients who might be in urgent need, due to restricted hospital beds or limited manufacturing slots, for example.

**Box 3:** Real world outcomes for patients with R/R LBCL treated with axicabtagene ciloleucel in  ≥ third-line in the Netherlands (Spanjaart et al., 2023)

In the Netherlands, patients with R/R LBCL were initially primarily treated with axi-cel, as it was the first reimbursed CAR-T product for R/R LBCL in ≥ 3^rd^-line. Below real world outcomes of patients with R/R LBCL treated with axi-cel in ≥ 3^rd^-line in the Netherlands are provided. These outcomes are comparable to other real world cohorts and the pivotal ZUMA-1 trial(Spanjaart et al., 2023).
*Efficacy* - Overall response rate (complete response rate): 84% (66%) - 12-month overall survival rate: 62% - 12-month progression-free survival rate: 48%
*Toxicity and hospital admission* - 12-month non-relapse mortality rate: 5% - CRS any grade (Grade ≥ 3): 92% (5%) - ICANS any grade (Grade ≥ 3): 62% (22%) - Any grade infections early (< 1 month) and late (≥ 1 months post-infusion): 26% and 43% - Severe (Grade ≥ 3) ongoing cytopenias at month 3 post-infusion: anemia (7%), neutropenia (34%), thrombocytopenia (15%) - Median hospital admission duration: 14 days (min–max: 7–78 days) - ICU admission rate and median ICU admission duration: 14%, 3 days (min–max: 1–19 days)
- ER visits and readmission between month 1–3 post-infusion: 18%

*Axi-cel* axicabtagene ciloleucel, *CAR-T* Chimeric antigen receptor T-cell, *CRS* Cytokine release syndrome, *ER* emergency room, *ICANS* Immune effector cell-associated neurotoxicity syndrome, *ICU* Intensive care unit, *LBCL* large B-cell lymphoma, *R/R* relapsed or refractory

After elucidating the central moral dilemma, the team asked questions to the case presenter in order to clarify the situation. One question that was asked was what the hematologist did to alleviate her doubts. She indicated that she consulted the available literature on CAR-T treatment in older patients such as Mr. Mulder. This patient was in good condition, and the literature indicated no clear evidence to withhold treatment in the case of Mr. Mulder (Berning et al., 2024; Johnson et al., 2024; Maakaron & William, 2023). Yet, especially since evidence on persisting and long-term side effects and impact on quality of life of patients in his age group was lacking at that time, she still had doubts. She also informed the group that she spoke to the patient about what was important to him. He indicated that he did not want to lose his independence; his autonomy was crucial to him. He also had a very strong wish to survive and described himself as a lively and active person. The case presenter however doubted whether he was fully aware of how side effects could affect exactly what he deemed important: his autonomy, independence, and quality of life, as CAR-T therapy may cause severe, long-term toxicity, reducing quality of life and independence.

Now that the dilemma and underlying doubts were clarified, the facilitator asked the participants to articulate what is important for them in the situation. The CAR-T treating hematologists and trained CAR-T nurse indicated that their primary priorities were ensuring the best chance of cure while avoiding harm from severe toxicity, as well as optimally informing their patient about all available treatment options, including comparisons of effectiveness, toxicities, impact on quality of life, and uncertainties regarding treatment outcomes and discussing the patient’s expectations to make a shared decision. They also valued respecting patient autonomy while not overburdening him with difficult decisions in a very stressful situation. The geriatrician mainly valued providing appropriate care by taking into account the wishes and values of the patient, as well as assessing and ensuring that the patient fully understands what a treatment decision entails. The CAR-T clinical trial coordinators and researchers mentioned that they mainly valued respecting the patient’s autonomy and taking into account the patient’s wishes in a best possible way, offering the best possible treatment in terms of cure, while also protecting the patient from severe and long-term side effects (i.e., not harming the patient), as well as increasing their medical knowledge on CAR-T treatment in older patients through cases such as that of Mr. Mulder.

After this exploration of the values, norms, and principles at stake in the case, the facilitator asked the participants to consider what they deemed *most* important in the situation, and what should be leading in taking action.

After a short reflection and discussion, the team agreed that it was morally justified that CAR-T therapy was provided. Leading in this conclusion was the strong and well-articulated wish of the patient to survive. This outweighed the possibility of (irreversible) harm. Yet, the possibility that the patient did not fully understand the consequences of this treatment choice was also acknowledged. The team agreed that they could have mitigated this risk by explaining the consequences much more in terms of what Mr. Mulder valued (his autonomy, his independence), and by emphasizing the considerable uncertainty regarding treatment outcomes.

The hematologist in training indicated that, if this would have been a patient who did *not* have such a strong wish for survival, and especially when there would have been more evidence that the chance of irreversible toxicity in this older patient group is higher, she might have refrained from offering CAR-T therapy. Furthermore, looking back, she concluded that she should incorporate a geriatric assessment during screening for patients aged  ≥ 70 years to reveal possible frailty and optimize the patient’s condition, and to clarify which values are most important for the patient and explicitly address these values while discussing all available treatment options (chance of cure, and the risk of toxicity, lengthy hospital admission and ICU admission, etc.).

During the evaluation of this MCD, the group indicated that they experienced the MCD as supportive, and that they expect it to help to make more deliberate choices in similar cases in the future. Sharing perspectives in a transparent and open way and joint dialogic reflection generated insights into what drives certain treatment choices and underlies perspectives on good care. The MCD was also argued to be conducive to team building, as it fosters learning from and understanding each other’s perspective. The case presenter described that discussing the case together with other colleagues reduced her uncertainty and moral distress regarding the choice she had made in the case of Mr. Mulder, which came as a relief. The participants agreed that organizing an MCD in anticipation of a difficult treatment decision could help to make more deliberate, well-considered choices. Additionally, it was mentioned that it could possibly be valuable to have the patient or relatives present at an MCD, although that would require more experience with these kind of ethical deliberations as a team. Finally, participants indicated that they would have preferred that the MCD was a bit shorter, especially in case you want to implement these sessions in routine clinical practice, for example as part of a multidisciplinary team meeting.

## Discussion

As CAR-T therapy continues to evolve, ethical reflection becomes increasingly important. The introduction of CAR-T therapy has not only transformed clinical practice but also raised complex ethical questions regarding patient selection, proportionality of risks, equity of access, and resource allocation (Bell et al., 2023; Imbach et al., 2018; Kourelis et al., 2023). Both Bell et al. and Kourelis et al. have highlighted the ethical challenges of equitable access to CAR-T therapy in the context of limited treatment capacity (Bell et al., 2023; Kourelis et al., 2023). Bell et al. underscored that fairness and equity demand a consistent approach that does not disadvantage medically underserved or underrepresented populations and described the application of Accountability for Reasonableness (A4R), a priority-setting framework grounded in procedural justice, to the problem of limited CAR-T slots in their institution. They identified four principal domains to consider in patient prioritization: expected medical benefit, risk of harm, psychosocial and contextual factors, and medical urgency. This framework provides a pragmatic template that can be adapted across institutions and may foster patient trust by ensuring decisions are reasonable, transparent, and open to appeal (Bell et al., 2023). Kourelis et al. conducted a survey across 17 centers in the United States and described the ethical challenges related to patient selection and prioritization with limited CAR-T manufacturing slots for patients with multiple myeloma and the procedures centers used to deal with this. For consistency in patient selection, all centers used a committee of experienced CAR-T physicians and some centers multidisciplinary teams, which sometimes also include nurses, social workers and ethicists. For transparency, 15 centers had selection criteria, timelines and priority scores available to CAR-T treating physicians. Core ethical values that were used when selecting patients for CAR-T therapy included maximize total benefit, treat people equally, give priority to the worst-off and promote/reward social value (e.g., younger patient first). They highlight the need for incorporating such ethical resource allocation strategies into formal policies of institutions to ensure equity of access (Kourelis et al., 2023). Considering the complexity of patient selection/prioritization and its dynamics as more experience is gained, transparent reporting, evaluating and reflecting upon these procedures, including who eventually receives CAR-T therapy, who does not, and reasons for (in)eligibility, is important to secure equitable and ethical CAR-T care.

Further research into the ethical dimensions of CAR-T therapy is warranted, not only in (hematologic) malignancies but also, as its indications expand, into non-oncologic disorders such as autoimmune diseases. It remains to be seen whether similar ethical challenges will emerge in these new contexts, underscoring the need for continued ethical dialogue alongside medical innovation.

Within specialized CAR-T units, structured MCDs can serve as a valuable tool to facilitate reflection and shared decision-making in ethically challenging situations and support healthcare professionals in choosing the right course of action. It facilitates a dialogue about what is ‘right’ in a specific case by giving time and space to elucidate the perspectives from all relevant stakeholders involved and to come to a well-founded moral judgment on the situation at hand. This can help clinicians in clinical decision-making, or to consider what they may do different in a future similar situation (Inguaggiato et al., 2019).

Moreover, if a series of MCDs is conducted on a specific theme and various perspectives and practical recommendations emerge from these sessions, this can help to complement guidelines and policy (Tan et al., 2018). It should be noted, however, that during the MCD discussed here, the institutional and societal perspective were not taken into consideration by the participants. Integrating perspectives from entities like hospital boards or national health funding authorities could however offer valuable insights into the (justification of) possible courses of action. This especially applies to institutional/national policy concerning resource allocation.

Overall, the participants evaluated the MCD as highly relevant and helpful. By providing a platform for in-depth discussion and reflection, it facilitated a better understanding of the decision that had been made and reduced moral distress. Furthermore, it enhanced moral competencies by encouraging thoughtful analysis of the ethical dilemma and was conducive to team building. These reported effects are in line with findings of evaluation studies on MCD (Molewijk et al., 2008; Tan et al., 2018). Besides, the concrete recommendations that emerged from the MCD were directly implemented in clinical practice and improved care and decision-making for older patients potentially eligible for CAR-T. Furthermore, conducting MCD in real-time during difficult situations, rather than solely in hindsight, was seen as potentially beneficial for supporting decision-making in the “heat of the moment”.

However, implementation of clinical ethics support such as MCD in routine clinical practice can be challenging. It requires a positive attitude (i.e., recognition of usefulness) of healthcare professionals towards ethics and joint reflection, and a trained facilitator, who might not always be available. Engaging healthcare professionals by training them to become MCD facilitators is found to increase the capacity for organizing and guiding MCDs, and to help to integrate MCD into clinical practice (Plantinga et al., 2012). Also, methods such as the dilemma method can be perceived as (too) time-consuming. Alternative and shorter methods besides the dilemma method exist and could provide a solution to this (Metselaar et al., 2022).

There are several options to implement MCD in clinical practice. For example by organizing a monthly MCD session with the entire (local and/or national) CAR-T team, which could also be an extension of an already existing clinical (multidisciplinary team) meeting. Additionally, MCD could be offered as an ad hoc consultation service (i.e., “ethics on call”).

Fostering a culture of ethical reflection and collaboration ensures that ethical considerations remain central to the delivery of CAR-T therapy. Thereby, clinical ethics support will enhance quality of patient care, uphold the ethical principles that underpin medicine, and contribute to the well-being of the medical team.

## Conclusions

CAR-T therapy represents a major advancement in the treatment of relapsed or refractory B-cell malignancies, offering survival benefits where few options previously existed. However, as we have discussed, its complexity, high costs, limited availability, risk of severe toxicity, the absence of standardized selection and prioritization criteria, and difficulty to fully predict outcomes for individual patients upfront create substantial clinical and ethical challenges. These challenges can generate moral distress, with implications for both professional well-being and the quality of patient care. To address these issues, structured clinical ethics support, particularly through MCD, may be implemented in clinical practice. By facilitating collective reflection, dialogue, and transparency, clinical ethics support can help clinicians navigate ethical uncertainty more confidently, support fair and patient-centered decision-making, improve quality of patient care, and strengthen team resilience in the evolving and ethically complex field of CAR-T therapy.

## Data Availability

All data generated or analysed during this study are included in this published article.

## Footnotes

1 Cases described in this article are fictional or have been anonymized.
